# Genome-Wide Identification and Salt Tolerance Analysis of the *SKS* Gene Family in Soybean

**DOI:** 10.3390/ijms27062522

**Published:** 2026-03-10

**Authors:** Fanglei He, Qiulian Lu, Weijie Hu, Weiliang Chen, Jianping Zhai, Jingyu Wu, Shuhan Chen, Ting Liao, Ziqi Zhu, Sisi Zheng, Chao Fang, Lingshuang Wang

**Affiliations:** Guangdong Key Laboratory of Plant Adaptation and Molecular Design, Innovative Center of Molecular Genetics and Evolution, School of Life Sciences, Guangzhou University, Guangzhou 510006, China; hefanglei@e.gzhu.edu.cn (F.H.); luqiuqiu@e.gzhu.edu.cn (Q.L.); 13556172449@163.com (W.H.); 13611466122@163.com (W.C.); zhaijianping@e.gzhu.edu.cn (J.Z.); wujingyu0503@163.com (J.W.); m15602702457@163.com (S.C.); 13622451244@163.com (T.L.); zzq@e.gzhu.edu.cn (Z.Z.); 13546875199@163.com (S.Z.)

**Keywords:** soybean, multicopper oxidase, *SKS*, gene family, salt stress

## Abstract

The *Skewed5 Similar* (*SKS*) genes play a crucial role in plant growth and development, as well as in responding to abiotic stress, by regulating cell wall remodeling and maintaining reactive oxygen species (ROS) homeostasis. The *SKS* gene family has not yet been thoroughly studied in soybean. We conducted a comprehensive genome-wide analysis of 88 *GmSKS* genes, systematically elucidating their gene structures, conserved protein domains, collinearity relationships, and phylogenetic relationships, to identify potential candidate genes associated with soybean tolerance to salt stress. The *GmSKS* genes are distributed across 18 chromosomes, and the expansion of this gene family is primarily attributed to the combined effects of tandem duplications and segmental duplications. Different tissue-specific expression patterns among *GmSKS* members were identified using expression profiling. Analysis of *cis*-regulatory elements further revealed that the promoter region may be involved in plant hormone signaling pathways and responses to abiotic stress. Furthermore, quantitative reverse transcription PCR (qRT-PCR) analysis showed that 21 of the 22 examined *GmSKS* genes were significantly upregulated under salt stress, while one was significantly downregulated. This expression pattern may be linked to salt tolerance mechanisms in soybean under stress conditions. Haplotype and selection trend analyses of *GmSKSs* revealed that varieties carrying *GmSKS5*^1123*G*^, *GmSKS22*^1727*G*^, *GmSKS43*^50*T*^ and *GmSKS71*^1213*T*^ are highly enriched in cultivated soybeans and have undergone artificial selection. This study provides basic information for the identification of salt stress-responsive gene resources of *GmSKS* family genes, and provides novel theoretical insights for the functional identification and cloning of soybean salt tolerance-related genes.

## 1. Introduction

Abiotic stresses have a range of negative impacts on plants that significantly limit their growth, development, and production. One of the most serious abiotic factors influencing agricultural output worldwide is salinity stress, which is brought on by an excessive amount of salt in the soil [1]. Upon receiving salt stress signals, plants initiate a series of complex adaptive responses. Excessive Na^+^ buildup, changes in intracellular Ca^2+^ concentration, and the quick generation of reactive oxygen species (ROS) are among the early signaling processes brought on by salt stress [2]. ROS are the byproducts of redox reactions that take place during aerobic respiration and photosynthesis, possessing potent oxidizing capabilities and serving as an indispensable component of plant metabolic pathways [3]. At low concentrations, they act as important signaling molecules in many physiological pathways [4]. Plants produce ROS as signaling molecules under abiotic stress. An excess of ROS and its byproducts can damage cellular structures, impair enzyme performance, cause lipid peroxidation, and ultimately lead to cell death [5]. The balanced regulation of ROS production and clearance is essential for many developmental processes in plants [6]. Plants need a complex antioxidant defense mechanism with both enzymatic and non-enzymatic components to combat oxidative damage brought on by salt. By eliminating excess ROS, the coordinated ROS-scavenging machinery effectively lowers oxidative damage and safeguards normal growth and developmental processes in saline settings [7,8,9,10,11,12].

The *Skewed5 Similar* (*SKS*) gene family is highly expressed in both monocotyledons and dicotyledons and belongs to a subfamily of *multicopper oxidase* (*MCO*) genes [13]. Multicopper oxidases are a class of proteins that bind copper (Cu^2+^) and are found in a wide range of organisms, including bacteria, fungi, plants, and animals. They are characterized by possessing four copper ions organized into three functional copper-binding centers: Type 1 (T1), Type 2 (T2), and Type 3 (T3). The T2 and T3 centers jointly form a trinuclear copper cluster that catalyzes O_2_ reduction [14,15]. Laccases and ascorbate oxidases (AOs) are the two main MCOs found in plants [16]. The *SKS* gene family was first identified in *Arabidopsis*, with *Skewed5* (*SKU5*) as its founding member. *SKU5* encodes a glycosylphosphatidylinositol (GPI)-anchored glycoprotein, which is localized to the plasma membrane and cell wall [13]. Although SKS proteins retain partial conservation of multicopper oxidase (MCO)-related domains, most members of this family lack the classic copper-binding motif and therefore typically lack classical oxidase activity. They are more likely to function through protein–protein interactions, thereby regulating cell wall remodeling, polar growth, and responses to abiotic stress [13]. The functional diversity of the *Arabidopsis SKS* gene family is vividly demonstrated through phenotypic analysis of mutants. For instance, the *atsku5* mutant exhibits a skewed and helical root phenotype [13]. Additionally, *AtSKS1*, *AtSKS3*, and *AtSKU5* are expressed in seedling root tissues, regulating cell polarity elongation and cell wall synthesis. Their knockout mutants exhibit shorter roots, irregularly shaped root cells, and thicker cell walls [17]. Functional loss of *AtSKS6* leads to abnormal vascular patterns in cotyledons [18]. Overexpression of *AtSKS13* enhances plant defense against insects, while *atsks13* mutants exhibit defects in vegetative growth, pollen development, and pollen tube formation [19]. Furthermore, *SKS* genes indirectly support the preservation of ROS balance during developmental processes and stress responses. For instance, the *atsks11/sks12* double mutant displays diminished ROS accumulation in pollen tubes [20]; in maize, loss of *ZmSKS13* function leads to excessive ROS accumulation, resulting in DNA damage and metabolic disruption, ultimately causing defective endosperm development, indicating that *ZmSKS13* maintains normal kernel development by modulating ROS homeostasis [21]. *SKU5* and *SKS1* are cell surface ROS regulators that control cell wall structure and root cell growth in *Arabidopsis*. Loss of *SKU5* and *SKS1* function results in abnormal cell division planes, outward bulging of the cell wall, ectopic iron deposition, and attenuated NADPH oxidase-dependent ROS overproduction at the epidermis-cortex and cortex-endodermis junctions in roots [22].

The domestication process involves both conscious and unconscious artificial selection, leading to dramatic morphological and physiological changes between domesticated plants and their wild ancestors [23]. Domestication, the introduction of genes from wild cousins, and environmental adaptation are the origins of modern crops. How ancient humans choose domestication traits and the origins of crops can be better understood by identifying domestication-related genes and their genetic diversity [24]. Soybean (*Glycine max*) is one of the most important crops globally and serve as a primary source of plant protein and oil. Severe soil salinization significantly reduces crop yield and quality [25,26]. Despite being classified as moderately salt-tolerant, soybean production and cultivation expansion remain limited by saline–alkaline stress [27]. Multiple salt stress-responsive genes have been identified in soybean, and specific allelic variants of these salt-tolerance genes underwent artificial selection during domestication. Natural variation in the dehydration-responsive element-binding (DREB) genes *DREB3a* and *DREB3b* is associated with differential salt tolerance in soybean. Notably, *DREB3b* appears to have undergone both natural and artificial selection. Compared to the reference allele (*DREB3b^Ref^*) in cultivated soybean, the wild soybean-derived allele *DREB3b*^39*Del*^ confers enhanced salt tolerance. The identification of this favorable *DREB3b* allele provides valuable genetic insight for improving soybean yield under saline conditions [28].

Currently, the evolutionary and functional roles and salt-responsive expression of the *SKS* gene family in soybean have not been systematically investigated. Here, we identified 88 *GmSKS* genes, characterized their structural and bioinformatic features, and profiled their transcriptional responses to salt stress via quantitative reverse transcription PCR (qRT-PCR). Based on this foundation, we analyzed the haplotypes and selection patterns of *GmSKS* using soybean resequencing data to identify key natural *GmSKS* variants under artificial selection. Our findings lay a crucial groundwork for functional dissection of the *GmSKS* gene family and for molecular breeding efforts targeting salt tolerance in soybean.

## 2. Results

### 2.1. Identification and Characterization of the SKS Family Members in Soybean

**Figure 1:**
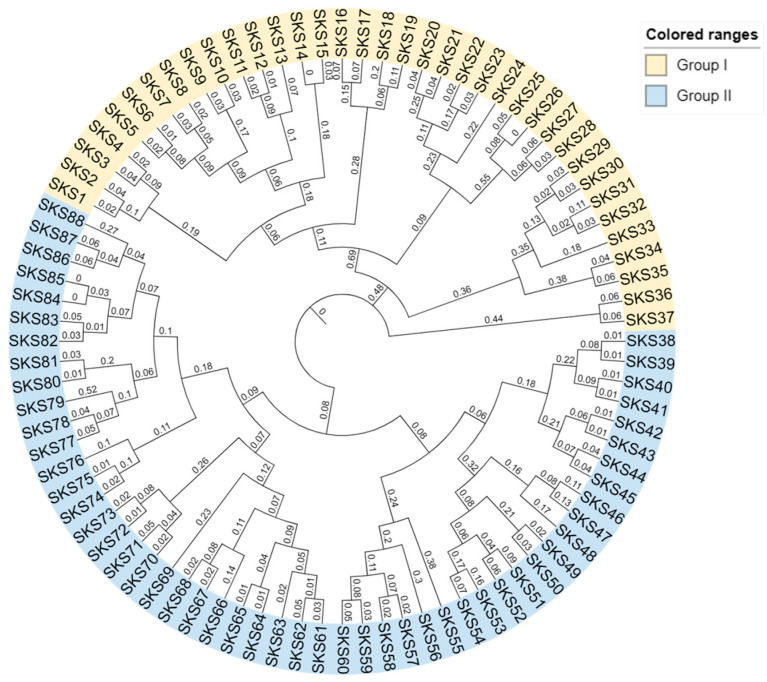
Phylogenetic relationships of *SKS* family genes from soybean.

To identify and characterize members of the *GmSKS* gene family, BLASTP analysis was performed using *Arabidopsis* thaliana protein sequences as query sequences. A total of 88 high-confidence *GmSKS* gene family members were identified, with their protein domain architectures predicted using the Pfam database (http://pfam.xfam.org/, accessed on 15 December 2025) and used to confirm their classification within the *GmSKS* gene family. A phylogenetic tree of the *GmSKS* gene family was constructed using maximum likelihood analysis (Figure 1).

The results indicate that the *GmSKS* gene family has undergone diversification during evolution. The *GmSKS* gene family includes 88 genes classified into two clades: Clade I (*GmSKS1*–*GmSKS37*; 37 genes) and Clade II (*GmSKS38*–*GmSKS88*; 51 genes). The physicochemical properties of the *GmSKS* gene family are summarized in Schedule S2. The shortest and longest proteins in the family are *GmSKS40* (388 aa; 43.1 kDa) and *GmSKS20* (637 aa; 71.7 kDa), respectively. GmSKS proteins exhibit pI values from 4.95 (*GmSKS26*) to 9.96 (*GmSKS75*), an instability index from 26.00 (*GmSKS85*) to 47.99 (*GmSKS27*), and an aliphatic index from 74.84 (*GmSKS27*) to 101.41 (*GmSKS62*).

### 2.2. Distribution and Collinearity Analysis of the GmSKS Genes

**Figure 2:**
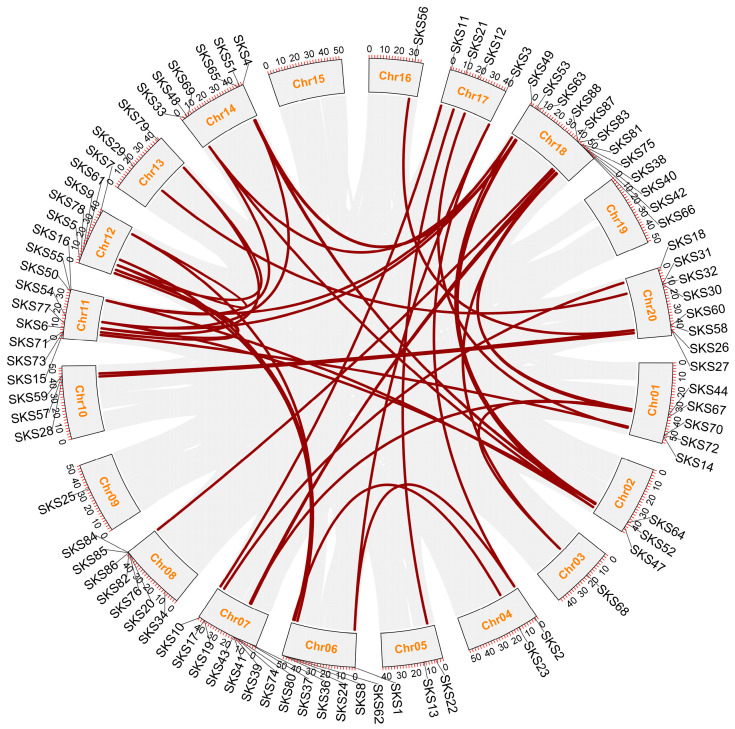
Chromosomal distribution and interchromosomal relationships of *GmSKSs.* Red curves connect pairs of genes with segmental duplications.

The *GmSKS* gene family is distributed across nearly all chromosomes (Figure 2), indicating that this gene family has undergone significant expansion within the soybean genome. Chr03, Chr09, and Chr16 of the soybean chromosomes each carry one *GmSKS* gene, whereas Chr04, Chr05, and Chr13 each have two genes. Chr02 and Chr10 each contain three *GmSKS* genes, while Chr18 contains the largest number, totaling twelve *GmSKS* genes. Chromosomes 15 and 19 did not have any *GmSKS* genes mapped to them. Most *GmSKS* genes are situated close to the ends of chromosomes, and their quantities differ significantly from one chromosome to another.

Tandem duplication and segmental duplication are key mechanisms for gene family expansion. While segmental duplication produces vast duplicated chromosomal portions within the genome and is frequently linked to chromosomal recombination, tandem duplication usually entails the appearance of two neighboring genes on the same chromosome [29,30]. We systematically analyzed the members of the *GmSKS* gene family based on collinearity analysis in order to explore the evolutionary origin and expansion process of this gene family. The results show that tandem duplication is highly enriched on several chromosomal locations, including Chr07, Chr08, Chr11, and Chr18. These chromosomes include several *GmSKS* genes that are physically grouped to form unique gene clusters. This pattern implies that the recent growth of this gene family is mostly driven by local tandem duplication. Furthermore, several pairs of chromosomes (such as Chr02-Chr14, Chr06-Chr12, and Chr07-Chr18) include large syntenic gene pairs that are joined by dense syntenic links to form distinctive syntenic blocks. These collinearity relationships closely match the characteristics of the paleopolyploidization (WGD) event that occurred in soybeans, indicating that large-scale segmental duplications mediated by WGD were the main cause of the early development of the *GmSKS* gene family.

We examined the Ka/Ks ratios (non-synonymous substitution rate to synonymous substitution rate) for 46 homologous *GmSKS* gene pairs in order to investigate possible selective forces causing duplication events. We obtained 34 valid estimates after excluding gene pairings with zero synonymous substitutions, saturated Ks values (Ks > 2), or poor codon alignment quality (Schedule S3). The results indicate that the Ka/Ks ratios for most *GmSKS* homologous gene pairs are significantly less than 1, indicating that these genes have undergone intense selective pressure during soybean evolution.

### 2.3. Genetic Structure and Conserved Domain Characteristics Encoded by the GmSKS Genes

**Figure 3:**
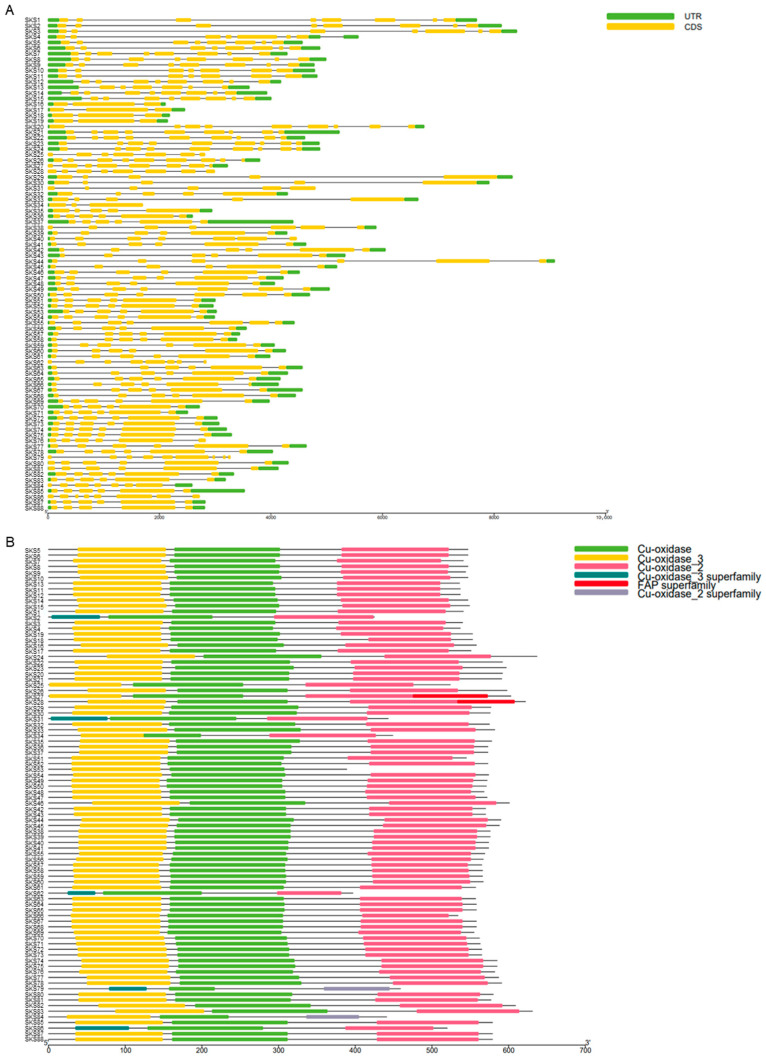
Structure of *GmSKS* gene family members; (**A**) gene structure of the *GmSKS* genes; (**B**) conserved domain of the GmSKS proteins.

Genetic structure analysis indicates that the number of exons and introns within the *GmSKS* gene family varies, ranging from a minimum of 3 exons and 2 introns to a maximum of 9 exons and 8 introns. The number and distribution of exons and introns are similar among genes within the same evolutionary clade. In evolutionary branch I, *GmSKS1*-*2*, *GmSKS4*, *GmSKS5*-*8*, *GmSKS12*, *GmSKS14*-*15*, and *GmSKS27*-*28* all contain 8 exons and 7 introns. *GmSKS3*-*4*, *GmSKS10*-*11*, *GmSKS13*, and *GmSKS25* each contain 7 exons and 6 introns, while *GmSKS9*, *GmSKS20*-*24*, *GmSKS26*, and *GmSKS25* each contain 9 exons and 8 introns, exhibiting extensive interspersed distribution across the genome. In evolutionary branch II, *GmSKS36-37*, *GmSKS39*, *GmSKS41-48*, *GmSKS51-54*, *GmSKS63-78*, *GmSKS80-83* and *GmSKS85* all contain 6 exons and 5 introns, accounting for approximately half of all members within the *GmSKS* gene family (Figure 3A). Members of the *GmSKS* gene family frequently have several conserved functional domains, according to structural domain analysis of 88 *GmSKSs* utilizing the Pfam database (http://pfam.xfam.org/search, accessed on 20 December 2025). Crucially, Cu-oxidase_3, Cu-oxidase, and Cu-oxidase_2 are the three essential copper oxidase-related domains found in each GmSKS protein (Figure 3B).

### 2.4. Analysis of Cis-Acting Elements in the GmSKS Promoters

**Figure 4:**
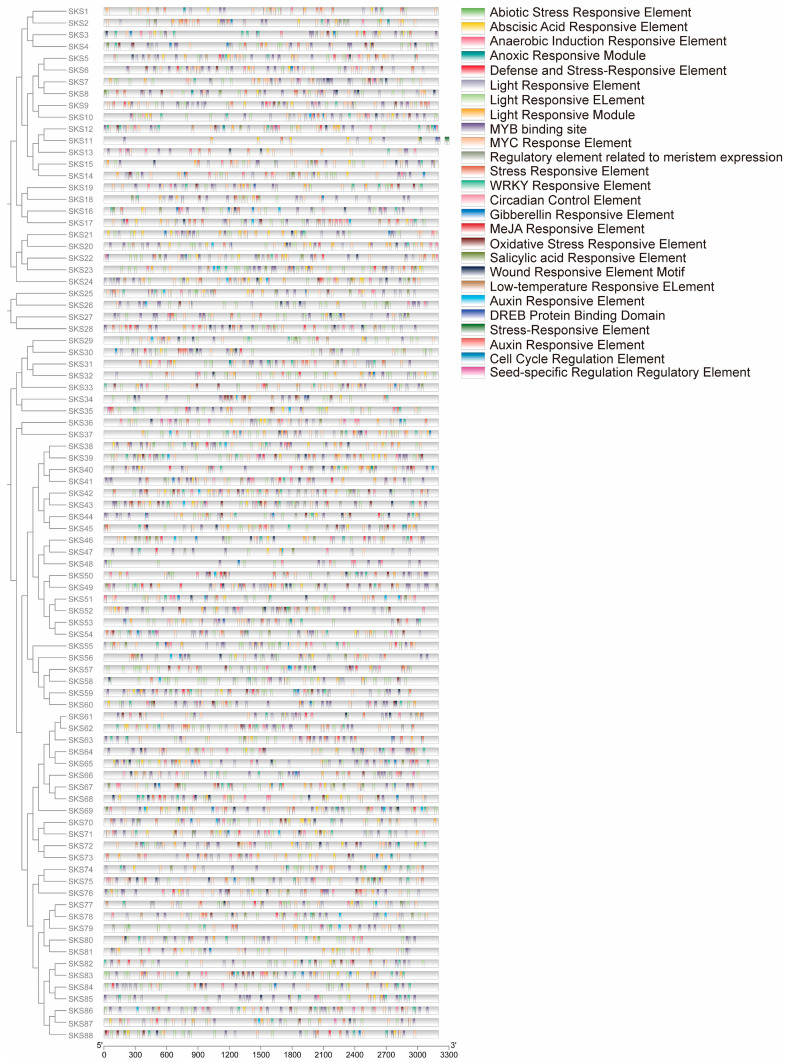
*Cis*-element analysis of *GmSKSs* promoters. The different types of *cis*-elements are represented by different colors. The length of the promoter sequence is indicated by scale bars at the bottom of the figure.

*Cis*-acting elements play a crucial role in the transcriptional regulation of gene expression. This study utilized the 3000 bp upstream sequence of each *GmSKS* genes to predict *cis*-acting elements. A total of 26 *cis*-regulatory elements were identified in the promoter regions of *GmSKS* genes. Among them, the *GmSKS* genes may be involved in light signaling due to the presence of circadian control elements and light-responsive elements in the promoter. Promoters also contain elements such as the Seed-specific Regulation Regulatory Element, the Regulatory Element Related to Meristem Expression, and the Cell Cycle Regulation Element. Furthermore, the most abundant elements are those associated with abiotic stress and plant hormones, such as the defense and stress-responsive element, abiotic stress-responsive element, anaerobic induction responsive element, low temperature-responsive element, oxidative stress-responsive element, auxin-responsive element, abscisic acid-responsive element, gibberellin-responsive element and MeJA-responsive element. The extensive and widespread distribution of *GmSKS* genes indicates their involvement in diverse biological processes and their distinct responses to abiotic stresses (Figure 4).

### 2.5. Expression Pattern of the GmSKS Genes

**Figure 5:**
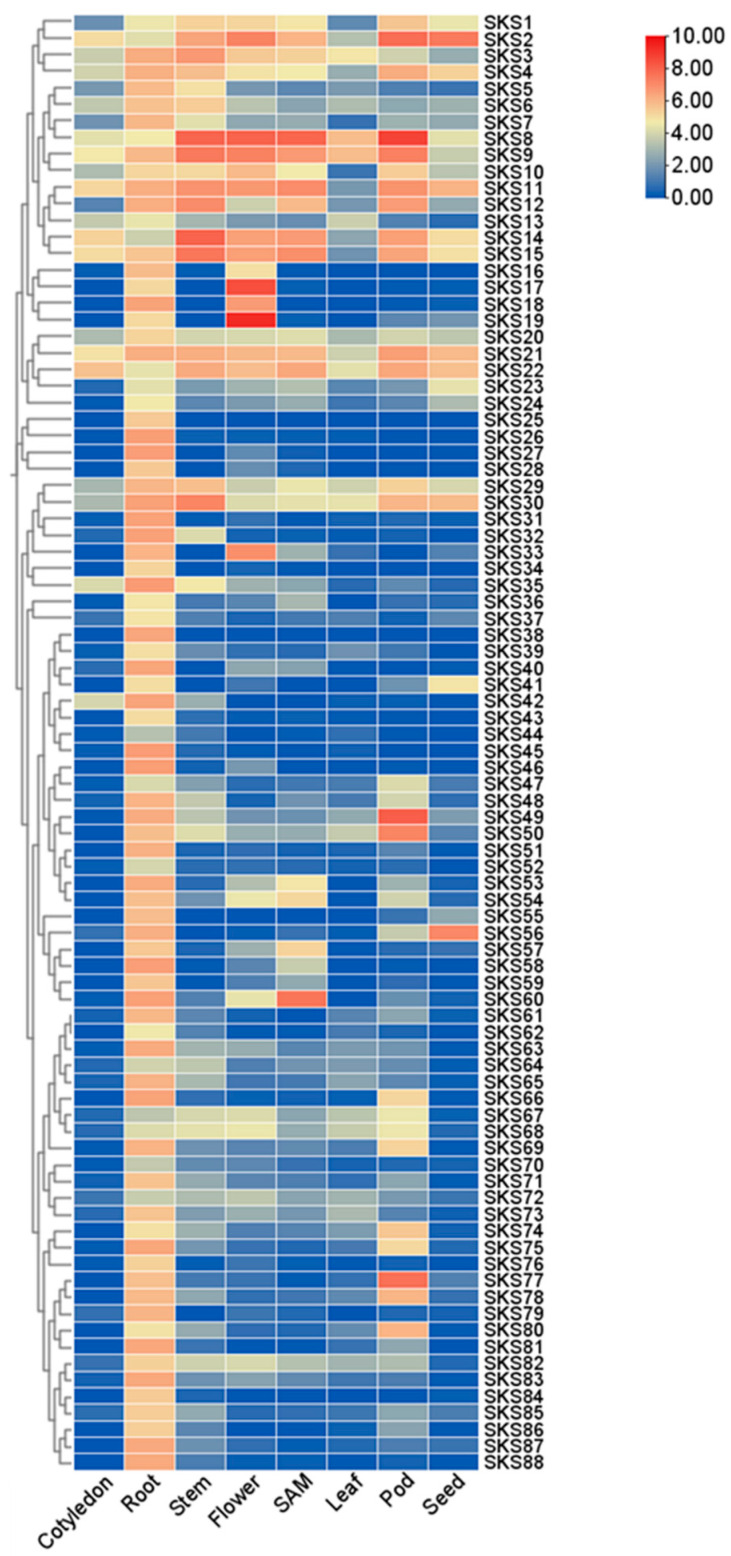
Expression analysis of *GmSKSs* in different tissues. Different colors represent log-transformed FPKM values; the gradient from blue to red indicates progressively increasing expression levels. Tissue-specific expression patterns of *GmSKS* genes were retrieved from the Soybean Multi-omics Database (SoyMD; https://yanglab.hzau.edu.cn/SoyMD, accessed on 26 December 2025).

Gene expression pattern data from the soybean multi-omics database SoyMD indicate that most *GmSKS* genes exhibit low expression levels in roots, stems, leaves, cotyledons, shoot apical meristems, flowers, pods, seeds, and seed coat tissues. Almost all members of the *GmSKS* gene family exhibit relatively high expression levels in root tissue. *GmSKS8-9* and *GmSKS21-22* are constitutively expressed at relatively high levels in all examined tissues. *GmSKS4*, *GmSKS10-11*, and *GmSKS14-15* exhibit relatively high expression levels in other tissues, except for lower expression levels in leaves. A subset of *GmSKS* genes have significant expression in particular tissues: *GmSKS60* is highly expressed in root and growth points, and *GmSKS17* and *GmSKS19* are highly expressed in flowers. Interestingly, *GmSKS44* exhibited almost no expression in any tissue. The results indicate that distinct *GmSKSs* may perform specific functions in particular tissues, revealing that the *GmSKSs* family exhibits tissue-specific functions (Figure 5).

### 2.6. The Response of the GmSKS Genes Under Salt Stress

**Figure 6:**
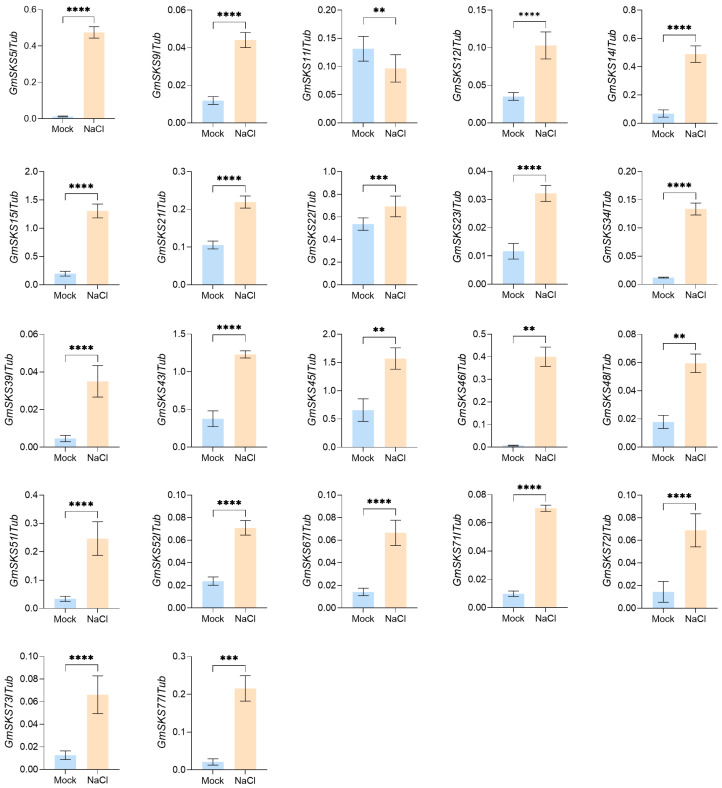
Quantitative real-time PCR expression analysis of 22 selected *GmSKSs* under salt stress. Expression levels were determined by qRT-PCR in root tissues of 15-day-old Williams 82 seedlings grown in Hoagland’s solution. The soybean *Tubulin* gene was used as reference control; error bars indicate standard deviation; asterisks denote significant differences in mean values compared to the unsalted control (Student’s *t*-test, ** *p* < 0.01, *** *p* < 0.001, **** *p* < 0.0001).

Previous research and expression analysis of *GmSKS* genes in plants indicate that these genes may be involved in stress responses and root growth. We used publicly available root transcriptome data from 15-day-old seedlings to screen and choose 22 highly expressed members for investigation in order to examine the regulatory dynamics of the *GmSKS* family during salt stress [31]. The expression levels of *GmSKS* genes in root tissues of 15-day-old soybean seedlings were compared between untreated (mock) and salt-treated (NaCl) root samples using qRT-PCR. The results showed that, after 4 h of salt treatment, the expression levels of 21 genes including *GmSKS5*, *GmSKS14-15*, *GmSKS34*, *GmSKS43*, *GmSKS51* and *GmSKS71* were significantly upregulated. In contrast, *GmSKS11* exhibited significant downregulation under the same salt treatment (Figure 6). These findings suggest that the *GmSKS* genes may play an important role in the early response to salt stress.

### 2.7. Haplotype and Domestication Selection Analysis of the GmSKS Genes

Analysis of gene haplotypes and the selection patterns of each haplotype is crucial for identifying superior alleles [32,33,34]. To investigate allelic diversity in the *GmSKS* gene family, we focused on a subset of *GmSKS* genes, analyzing their haplotypes and selection patterns during domestication and breeding. The study found that *GmSKS9*, *GmSKS11*–*12*, *GmSKS15*, *GmSKS23*, and *GmSKS51*–*52* exhibit almost no non-synonymous mutations, or the number of accessions carrying such mutations is extremely low. It is speculated that these genes may reside in heterochromatic regions, where mutations occur less frequently, or that their functions are highly conserved—such that mutations could impair soybean growth or reduce environmental adaptability (Supplementary Figure S1A–C,E,F,J,K). *GmSKS14*, *GmSKS34*, *GmSKS39*, *GmSKS48*, and *GmSKS67* exhibit multiple natural variants, with a considerable number of accessions carrying each variant. Among them, haplotypes such as *GmSKS14^H^*^2^, *GmSKS34^H^*^2^, and *GmSKS39^H^*^12^ have become increasingly enriched in cultivated soybean (Supplementary Figure S1D,G,H).

**Figure 7:**
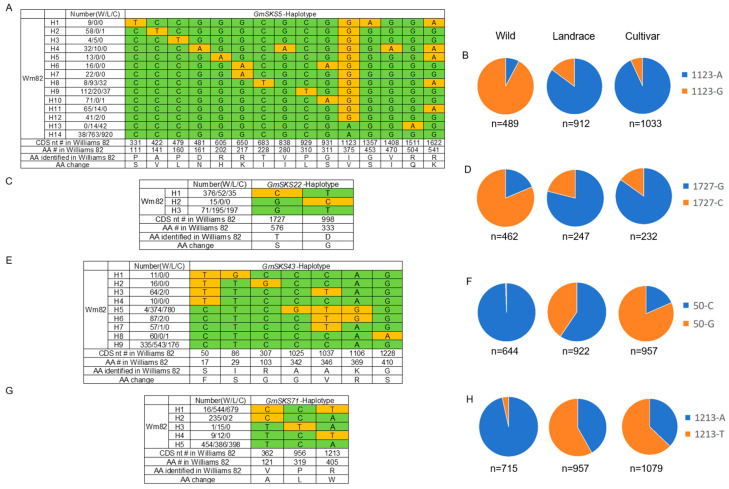
*GmSKS* genes were subjected to intense artificial selection. (**A**,**C**,**E**,**G**) represent the haplotypes of *GmSKS5*, *GmSKS22*, *GmSKS43*, and *GmSKS71*. (**B**,**D**,**F**,**H**) represent the proportion of different alleles in wild soybeans, farm varieties and cultivated varieties. Data were obtained from over 3000 sequenced accessions [35,36,37]. In the haplotype diagram, green highlights the reference allele as defined in the Williams 82 reference genome, and yellow highlights the alternate allele observed in the analyzed accessions. nt = nucleotide; # = position number in Williams 82.

Notably, the *GmSKS* gene family contains genes that have undergone intense artificial selection, including *GmSKS5*, *GmSKS22*, *GmSKS43*, and *GmSKS71*. In the coding sequences of *GmSKS5* and *GmSKS22*, a single predominant polymorphic site was identified. In *GmSKS5*, an A to G substitution at nucleotide position 1123 results in the replacement of isoleucine (I) with valine (V) at amino acid position 375. Wild soybean accessions predominantly carry the *GmSKS5*^1123*G*^ allele at this site. During soybean domestication and improvement, the frequency of *GmSKS5*^1123*G*^ gradually decreased, whereas the proportion of accessions carrying *GmSKS5*^1123*A*^ progressively increased, indicating that *GmSKS5*^1123*A*^ was preferentially selected during these processes (Figure 7A,B). In *GmSKS22*, a G to C substitution at nucleotide position 1727 results in the replacement of threonine (T) with serine (S) at amino acid position 576. Wild soybean accessions predominantly carry the *GmSKS22*^1727*C*^ allele at this site. During soybean domestication and improvement, the frequency of *GmSKS22*^1727*C*^ gradually decreased, while the proportion of accessions carrying *GmSKS22*^1727*G*^ progressively increased, indicating that *GmSKS22*^1727*G*^ was preferentially selected during these processes (Figure 7C,D). In *GmSKS43*, a C to T substitution at nucleotide position 50 leads to the substitution of serine (S) with phenylalanine (F) at amino acid position 17. Wild soybeans primarily harbor the *GmSKS43*^50*C*^ allele at this locus. Throughout domestication and breeding, the frequency of *GmSKS43*^50*C*^ steadily declined, whereas the frequency of *GmSKS43*^50*T*^ (derived from the C-to-T change) rose significantly, suggesting strong selection for *GmSKS43*^50*T*^ (Figure 7E,F). In *GmSKS71*, an A to T transversion at nucleotide position 1213 causes arginine (R) to be replaced by tryptophan (W) at amino acid position 405. The *GmSKS71*^1213*A*^ allele is predominant in wild soybean. However, its frequency markedly decreased during domestication and improvement, concomitant with a steady increase in accessions carrying *GmSKS71*^1213*T*^, demonstrating that *GmSKS71*^1213*T*^ was progressively favored by artificial selection (Figure 7G,H).

Our findings suggest that the majority of *GmSKS* genes play pivotal and evolutionarily conserved roles in soybean growth and development, exhibiting remarkable sequence stability. Nevertheless, the natural variations in *GmSKS5*, *GmSKS22*, *GmSKS43*, and *GmSKS71* have undergone intense artificial selection during soybean domestication and improvement. These variations may be associated with soybean adaptability under specific environmental conditions and human requirements.

## 3. Discussion

Salt stress impairs plant growth and physiology through multiple mechanisms, including ion imbalance, oxidative stress, and osmotic stress, among others. Plants may even die as a result of extremely high salt concentrations [38,39,40,41]. Plants rapidly respond to various stress conditions, and in many cases, signal transduction is linked to changes in ROS levels. By controlling ROS homeostasis, plant *SKS* preserves normal plant development and is essential for reacting to abiotic stress [19,21]. A set of genes that encode proteins related to polycopper oxidase and are extensively distributed in plants is represented by the *SKS* gene family. These genes, which were initially discovered in *Arabidopsis thaliana*, are involved in processes like cell wall remodeling, root geotropism, and cell elongation [17,22]. As of now, no study has reported the identification of *SKS* gene family members in soybean. This study leveraged the latest whole-genome sequencing data of soybean and *Arabidopsis SKS* gene family sequences to identify 88 *GmSKS* genes. Members within the same phylogenetic clade exhibit highly similar gene structures and conserved protein domains. Structural analysis revealed that all GmSKS proteins contain three highly conserved copper oxidase-related domains: the N-terminal Cu-oxidase_3 domain binds a Type 1 copper ion and serves as the primary site for substrate binding and electron acceptance; meanwhile, the central Cu-oxidase domain and the C-terminal Cu-oxidase_2 domain cooperatively provide ligands to assemble the trinuclear Type 2/Type 3 copper center. The latter effectively converts molecular oxygen (O_2_) to water and acts as the catalytic reaction’s active core. This catalytic unit serves as the structural foundation for the SKS family’s effective electron transfer from substrates to oxygen. It is modularly constructed from three domains. This implies that it may be involved in important physiological functions such as auxin polar transport, ROS homeostasis regulation, or cell wall remodeling. Furthermore, the persistence of these three core domains implies that the *GmSKS* gene family has preserved a unified molecular functional framework throughout its evolutionary history, despite slight differences in domain length or linking areas among individual members.

Genome duplication events are considered the primary cause of gene family expansion, with tandem duplication and segmental duplication being the predominant modes of replication. The results indicate that members of the *GmSKSs* gene family form distinct gene clusters through proximity on multiple chromosomes, each residing within 46 segmental duplication regions. The retention of tandemly duplicated *GmSKS* genes within syntenic blocks derived from whole-genome duplication (WGD) suggests that the family has evolved through layered duplication events. These include localized tandem amplification and large-scale genomic duplication, which together enabled subsequent subfunctionalization or neofunctionalization. A majority of *GmSKSs* exhibit tissue-specific expression patterns with varying expression levels, indicating they may perform distinct functions in different plant organs. *GmSKS17* and *GmSKS19* exhibit higher expression levels in flowers, implying their potential involvement in processes such as floral organ formation, pollen development, or flowering. *GmSKS60* exhibits high expression in the meristem, implying its potential involvement in regulating core meristem functions such as cell division, hormone signaling, or organ initiation.

This study explores *GmSKSs*’ potential role in soybean salt tolerance responses based on its expression pattern and existing literature support. This study utilized *cis*-acting element analysis within the promoter region to reveal that the *GmSKSs* promoter contains numerous *cis*-acting elements associated with responses to light, abiotic stress, and hormone induction. This indicates that the *GmSKS* genes may be involved in multiple biological processes. Under salt stress conditions, quantitative analysis of differential expression among 23 members of the *GmSKS* gene family revealed that 22 *GmSKSs* exhibited significantly upregulated expression, indicating that *GmSKSs* may positively regulate salt stress tolerance mechanisms. Moreover, studies have shown that *GmSKS11* is significantly downregulated under salt stress, implicating potential functional differentiation of the gene. ROS play a dual role in plants under salt stress: at moderate levels, they function as signaling molecules to activate defense pathways, but their excessive accumulation causes oxidative damage, including membrane lipid peroxidation and cell death [39]. Several *SKS* family genes have been implicated in modulating ROS homeostasis in plant. For instance, *AtSKS11*/*12* mutants exhibit reduced ROS in pollen tubes, *ZmSKS13* loss causes excessive ROS leading to defective kernel development in maize, and *AtSKU5*/*AtSKS1* act as cell surface regulators that maintain root growth and cell wall integrity by modulating NADPH oxidase-dependent ROS production [20,21,22]. Given that multiple *GmSKS* genes are strongly upregulated under salt treatment in soybean, and considering the conserved structural features of SKS proteins, including putative copper-binding domains suggestive of oxidoreductase activity, we hypothesize that *GmSKS* members may contribute to salt tolerance by ROS dynamics. Specifically, they may help maintain ROS at optimal signaling levels while preventing toxic overaccumulation, thereby preserving cellular integrity and sustaining growth under salt stress. In addition to its role in ROS homeostasis, abscisic acid (ABA) serves as a central regulator of salt tolerance in soybean. Numerous studies have demonstrated that ABA enhances salt tolerance in soybean by promoting the accumulation of antioxidants, scavenging ROS, and activating stress-responsive transcription factors [42,43,44]. Given the strong and rapid upregulation of multiple *GmSKS* genes observed under salt treatment, and considering that ABA signaling is a key early component of the salt stress response, it is plausible that their expression may be modulated either directly or indirectly by ABA. Future studies can aim to clarify this mechanism.

In temperate regions of China, cultivated soybeans were domesticated from its wild progenitor (*Glycine soja* Sieb. & Zucc.) approximately 5000 years ago [45]. Moreover, long-term artificial selection and population genetic bottlenecks have led to reduced genetic diversity, resulting in the loss of certain key genes or loci—particularly critical genetic variations associated with salt tolerance [28]. Identifying allelic variation within soybean germplasm resources and screening for essential mutation types contributes to enriching crop genetic diversity, providing a crucial genetic foundation for molecular breeding. This study reveals that natural variation in *GmSKS5*, *GmSKS22*, *GmSKS43*, and *GmSKS71* has undergone intense artificial selection during soybean domestication and improvement. Further analysis of these allelic variants will assist in breeding soybean varieties with high salt tolerance. Given that salt tolerance is not considered a domestication trait in soybean, it remains unclear why only *GmSKS5*^1123*G*^, *GmSKS22*^1727*G*^, *GmSKS43*^50*T*^ and *GmSKS71*^1213*T*^ were strongly selected during domestication. It is speculated that these genes may reside in genomic hotspots associated with multiple domestication and agronomic traits, such as flowering time, seed size, and plant height. Additionally, the alleles may have been co-selected when breeders chose for domestication-related genes since these genes may be genetically connected to loci governing important domestication features in soybeans.

In conclusion, the *GmSKS* gene family exhibits high structural conservation but possesses potential for functional diversification. By controlling cell wall characteristics and ROS signaling networks, the polycopper oxidase proteins that these genes produce probably play a critical role in salt stress responses. To date, no functional phenotypes of *SKS* family genes have been reported in soybean. Our results contribute preliminary evidence that may inform future studies on the roles of this gene family in legumes.

## 4. Materials and Methods

### 4.1. Plant Growth and Treatment

The soybean cultivar Williams 82 (Wm82) was used for gene expression analysis under salt stress. As a standard reference genotype with a fully sequenced genome, Wm82 is widely employed in soybean functional genomics research and is generally regarded as exhibiting moderate sensitivity to salt stress [46,47]. Healthy soybean seeds were germinated in vermiculite for 5 days and then transferred to culture pots containing Hoagland nutrient solution. The seedlings were grown in a Conviron growth chamber (Conviron, Winnipeg, MB, Canada) under long-day conditions (16 h light/8 h dark) at 25 °C, 60% relative humidity, and a light intensity of 500 μmol·m^−2^·s^−1^. When plants reached the VE growth stage (unifoliolate leaves fully expanded, approximately 15 days after sowing), the treatment group was subjected to 200 mmol·L^−1^ NaCl solution, while the control group was maintained in nutrient solution without NaCl. Root tissues were collected 4 h after NaCl treatment. Nine uniformly sized plants were grown under controlled conditions for each treatment. From these, three independent biological replicates (each consisting of three plants) were used for RNA extraction and subsequent gene expression analysis. Throughout the cultivation period, continuous aeration was provided using an air pump to ensure sufficient oxygen supply and support effective root respiration.

### 4.2. Identification and Annotation of the SKS Family Members in Soybean

Using *Arabidopsis* SKS protein sequences as queries, homologous genes in soybean were identified through BLASTP searches (https://blast.ncbi.nlm.nih.gov/, accessed on 15 December 2025) against the *Glycine max* genome (Phytozome v13; https://phytozome-next.jgi.doe.gov/, accessed on 15 December 2025). Stringent criteria were applied: a minimum amino acid identity of 60% and an E-value ≤ 1.0 × 10^−20^ [48]. Candidate proteins were further validated by domain annotation using the Pfam database (http://pfam.xfam.org/, accessed on 15 December 2025), confirming the presence of conserved SKS family domains [49].

### 4.3. Prediction of the Physicochemical Properties of GmSKS Proteins

Using the ProtParam tool from ExPASy (https://web.expasy.org/protparam/, accessed on 16 December 2025) to analyze the predicted soybean SKS proteins for their protein length, molecular weight, theoretical isoelectric point (pI), instability index and aliphatic index [50].

### 4.4. Phylogenetic Analysis of the GmSKS Genes

To investigate the phylogenetic relationships of *GmSKS* genes, multiple sequence alignment of the corresponding amino acid sequences of soybean and *Arabidopsis SKS* genes was performed using MEGA10 software. A maximum likelihood phylogenetic tree was constructed with MEGA10, supported by bootstrap analysis with 1000 replicates [51]. The tree was further visualized using the Interactive Tree Of Life (iTOL) platform [52].

### 4.5. Genetic Structure and Analysis of Conserved Domains in the GmSKS Genes

Using TBtools-II (version 2.135) to analyze the gene structure, exon and intron positions, and the number of *GmSKS* genes. Gene structure diagrams for the 88 members of the *GmSKS* gene family were generated using TBtools based on GFF3 annotation files. The conserved domains of their encoded proteins were predicted with the NCBI CDD (https://www.ncbi.nlm.nih.gov/cdd/, accessed on 20 December 2025) and subsequently visualized using TBtools [53,54].

### 4.6. Chromosome Localization and Collinearity Analysis of the GmSKS Genes

Obtain the physical chromosomal locations of *GmSKS* gene family members from the Phytozome database. Perform collinearity analysis of soybean internal structures using the One Step MCScanX plugin in TBtools, and visualize the results using the Advanced Circos plugin. To investigate the selection patterns experienced by *GmSKSs* following repetitive events, the ratio of non-synonymous (Ka) to synonymous (Ks) substitution rates for each *GmSKS* gene pair was calculated using the simple Ka/Ks calculator in MEGA10.

### 4.7. Cis-Acting Element Analysis of the GmSKS Genes

The 3 kb upstream sequences of each *GmSKS* gene were retrieved from genomic databases. Putative *cis*-regulatory elements within these regions were identified using the PlantCARE platform (http://bioinformatics.psb.ugent.be/webtools/plantcare/html/, accessed on 23 December 2025) and visualized with TBtools to show their types and distribution [55].

### 4.8. Analysis of the GmSKS Gene Expression in Different Tissues

Gene expression data were obtained from the soybean multi-omics database SoyMD (https://yanglab.hzau.edu.cn/SoyMD/#/transcriptomics/expression, accessed on 26 December 2025). The data cover five tissues: cotyledon, root, leaf, shoot apical meristem (SAM), stem, flower, pod, seed and seed coat. Heatmaps were generated to show the expression patterns of the *GmSKS* gene family across these tissues [35].

### 4.9. Analysis of the GmSKS Gene Expression Under Salt Stress

Candidate *GmSKS* genes were initially selected based on their high expression in publicly available RNA-seq data from 15-day-old soybean seedlings grown under control conditions. Details and accession numbers for these datasets are provided in reference [31].

Soybean seedling roots were harvested from 4 h following salt stress application, and total RNA was isolated using an UltraPure RNA Extraction Kit (CWBIO, Beijing, China). Using the Prime Script RT Reagent Kit with gDNA Eraser (Takara, Tokyo, Japan), reverse-transcribe 500 μg of RNA into cDNA. Using *GmTub* as the internal control gene, PCR reactions were performed using the real-time quantitative PCR kit (cat. no. RR430, Takara, Japan) on the Roche Light Cycler 480 instrument (Roche Molecular Biochemicals, Pleasanton, CA, USA). Each 10 µL reaction contains 1 µL of 1:5 diluted cDNA, 0.2 µL of each primer, 5 µL of SYBR Green Master Mix, and water to a final volume of 10 µL. Each sample was analyzed in triplicate, with three independent biological replicates. The primers used in this study are listed in Schedule S1. Relative expression changes of each *GmSKS* gene under salt stress were calculated using the 2^−ΔΔCT^ method [56]. All quantitative analyses were conducted using three independent biological replicates (each consisting of three plants). Data from all replicates were included in the statistical analysis, and results are presented as mean ± standard deviation. GraphPad Prism software (version 10.6.0, GraphPad Software, San Diego, CA, USA) was used for data visualization and to perform Student’s *t*-tests to assess significant differences between the control and experimental groups [57].

### 4.10. Haplotype and Domestication Selection Analysis

The vcftools program (version 0.1.16) was used to create SNP annotation files and genotyping data for the target gene across over 3000 materials based on the chromosomal position of the *GmSKS* genes. The target interval’s gene area was then categorized, with exonic sections chosen as the main emphasis. SNPs in this area were subjected to haplotype analysis in order to compare the compositions of cultivated, farmer-savored, and wild soybean.

## 5. Conclusions

This study identified 88 *GmSKS* genes, which exhibit structurally similar features within each phylogenetic clade. The *GmSKS* gene family is distributed across 18 soybean chromosomes, and its expansion has been shaped by both tandem and segmental duplications. A majority of *GmSKSs* exhibit a certain degree of tissue specificity. The promoter region of *GmSKSs* contains several *cis*-acting elements associated with responses to light, abiotic stress, and hormone induction. Of the numerous members comprising the *GmSKS* gene family in soybean, we focused on 22 that were highly expressed in root tissues. Under salt stress conditions, 21 genes exhibited upregulation, while *GmSKS11* exhibited downregulation. These genes may be involved in salt response in soybean. Varieties carrying *GmSKS5*^1123*G*^, *GmSKS22*^1727*G*^, *GmSKS43*^50*T*^ and *GmSKS71*^1213*T*^ are highly enriched in cultivated soybean and have undergone strong artificial selection.

## Data Availability

The original contributions presented in the study are included in the article/Supplementary Material. Further inquiries can be directed to the corresponding authors.

## Supplementary Materials

The following supporting information can be downloaded at: https://www.mdpi.com/article/10.3390/ijms27062522/s1.
