# Supplementary material for: Genome-Wide Identification and Salt Tolerance Analysis of the SKS Gene Family in Soybean
Source: Int J Mol Sci. 2026 Mar 10;27(6):2522. doi: 10.3390/ijms27062522 (PMC13026732; doi:10.3390/ijms27062522)
Supplement: Supplementary file 1 [file ijms-27-02522-s001.zip › Supplementary Figure.pptx]

## Slide 1
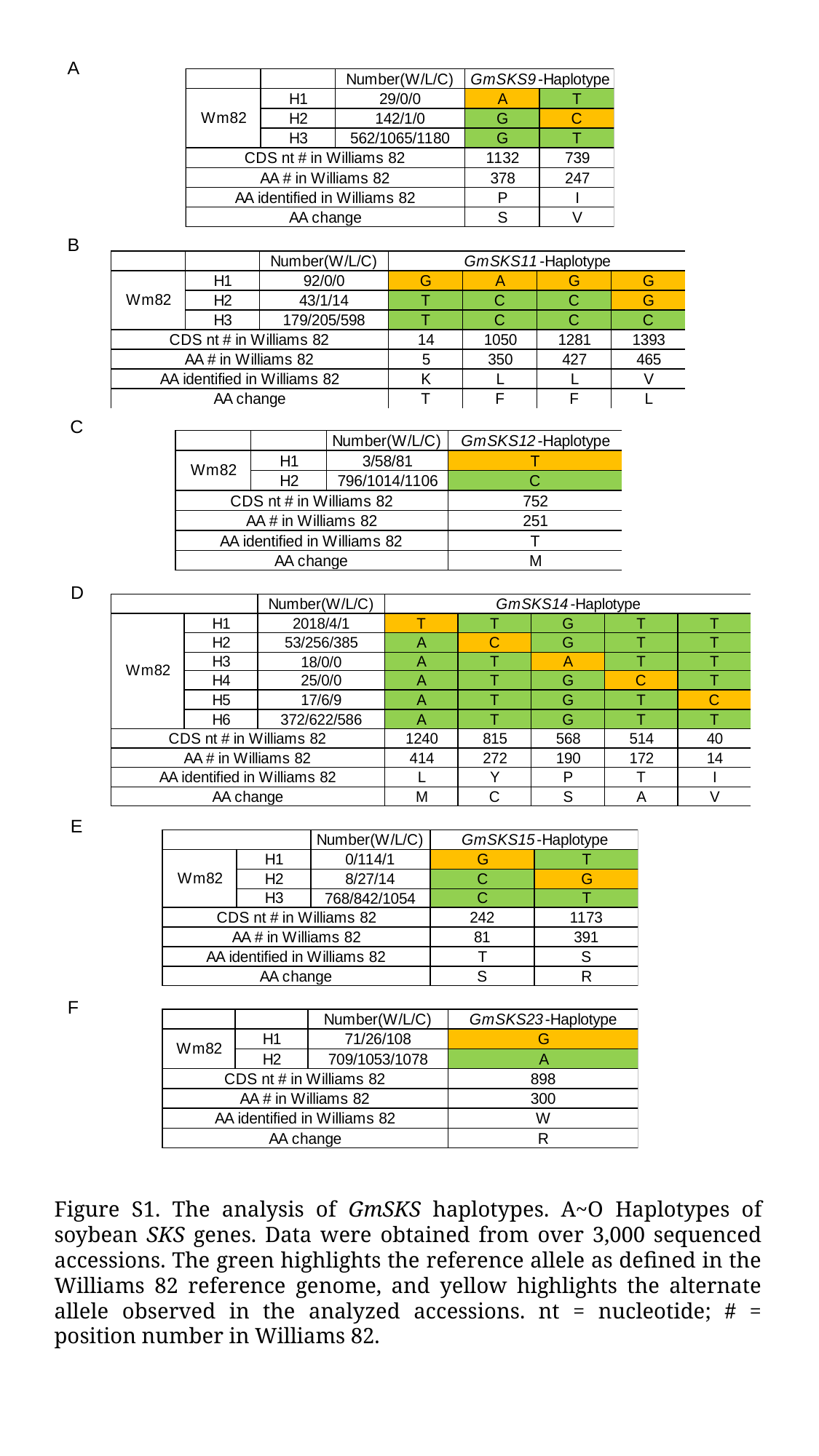

A
B
C
D
E
F
Figure S1. The analysis of GmSKS haplotypes. A~O Haplotypes of soybean SKS genes. Data were obtained from over 3,000 sequenced accessions. The green highlights the reference allele as defined in the Williams 82 reference genome, and yellow highlights the alternate allele observed in the analyzed accessions. nt = nucleotide; # = position number in Williams 82.

## Slide 2
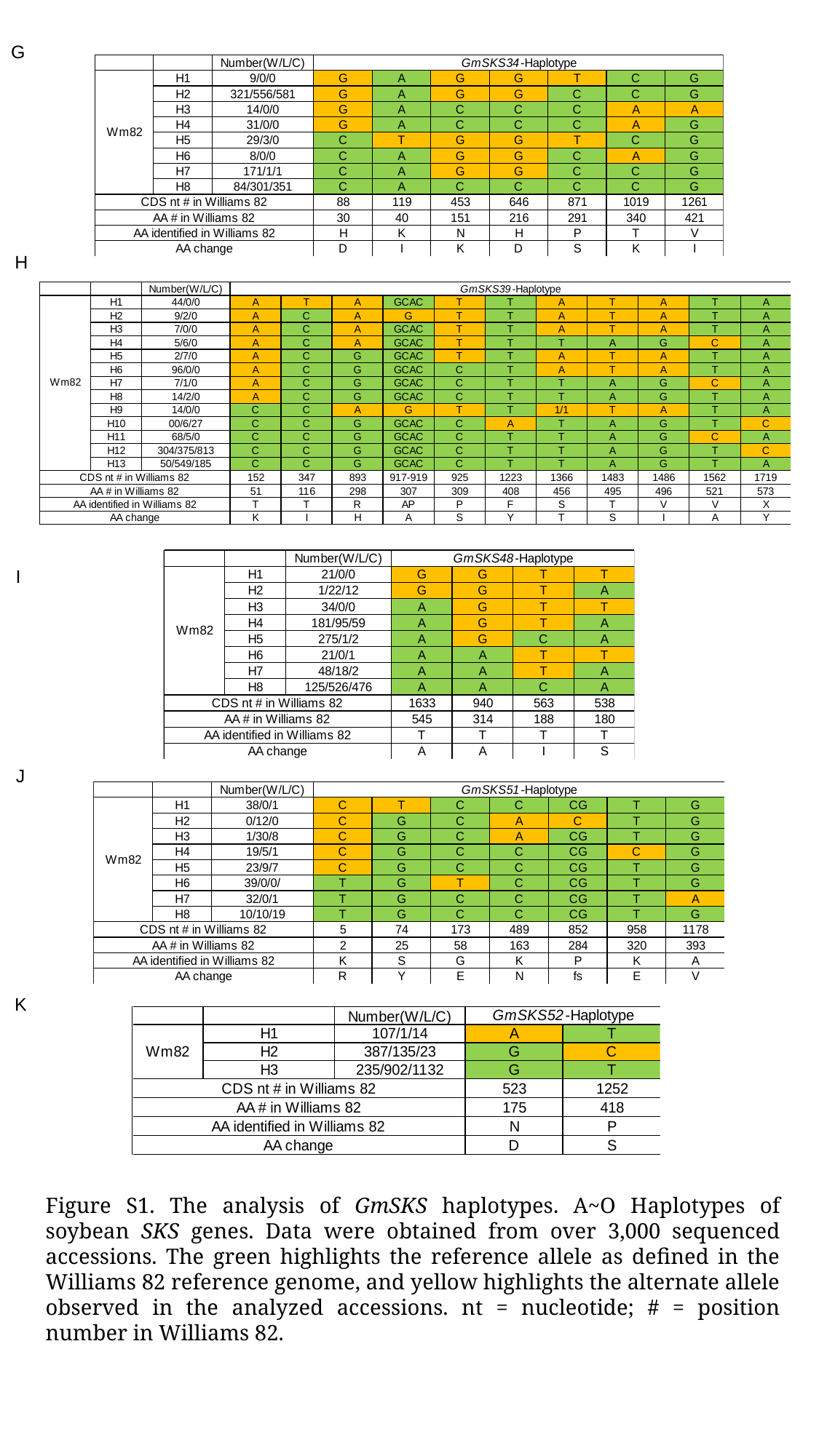

G
H
I
J
K
Figure S1. The analysis of GmSKS haplotypes. A~O Haplotypes of soybean SKS genes. Data were obtained from over 3,000 sequenced accessions. The green highlights the reference allele as defined in the Williams 82 reference genome, and yellow highlights the alternate allele observed in the analyzed accessions. nt = nucleotide; # = position number in Williams 82.

## Slide 3
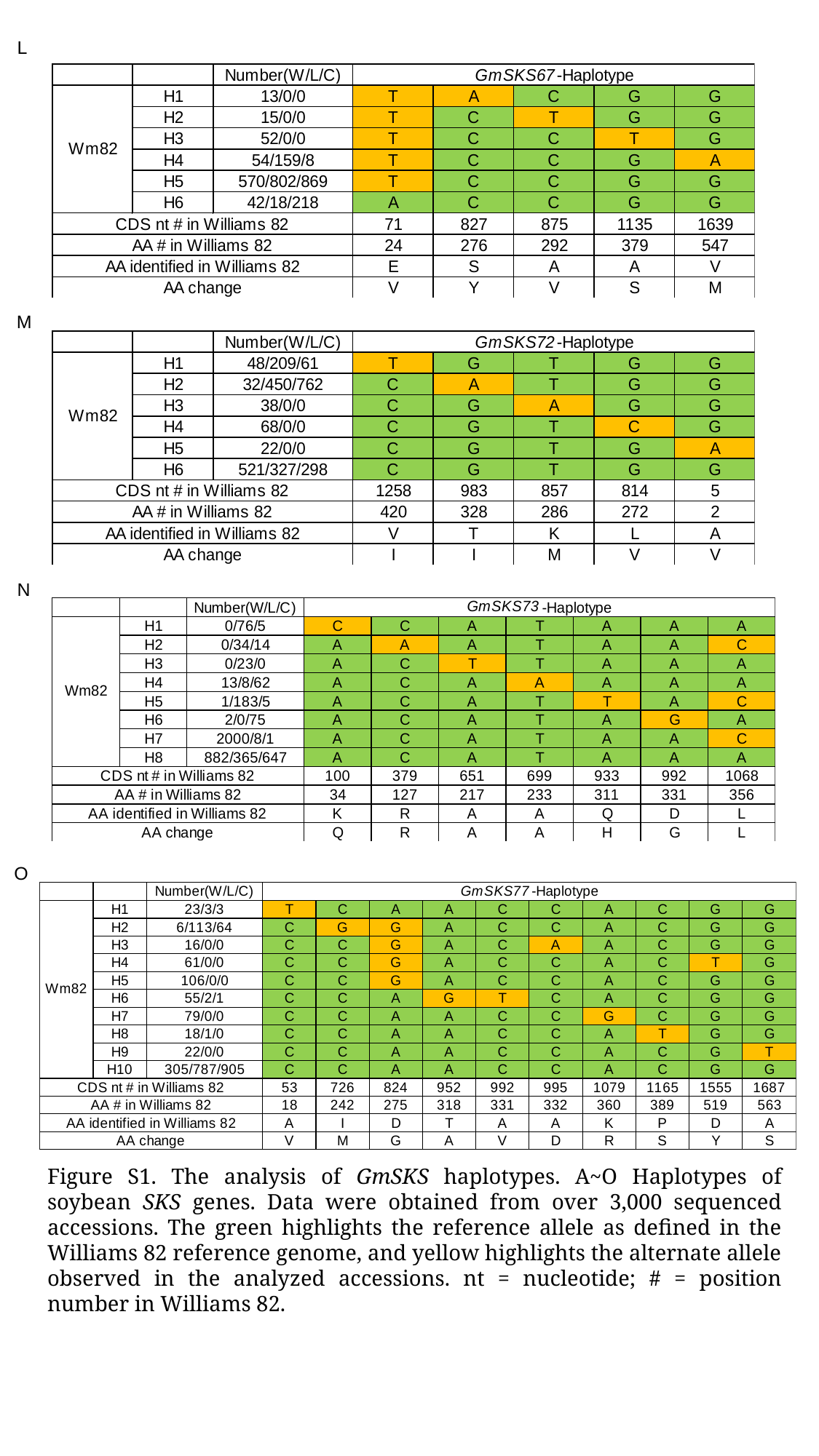

L
M
N
O
Figure S1. The analysis of GmSKS haplotypes. A~O Haplotypes of soybean SKS genes. Data were obtained from over 3,000 sequenced accessions. The green highlights the reference allele as defined in the Williams 82 reference genome, and yellow highlights the alternate allele observed in the analyzed accessions. nt = nucleotide; # = position number in Williams 82.
